# Dimensions of Impulsivity in Gambling Disorder

**DOI:** 10.1038/s41598-019-57117-z

**Published:** 2020-01-15

**Authors:** Gemma Mestre-Bach, Trevor Steward, Roser Granero, Fernando Fernández-Aranda, Teresa Mena-Moreno, Cristina Vintró-Alcaraz, María Lozano-Madrid, José M. Menchón, Marc N. Potenza, Susana Jiménez-Murcia

**Affiliations:** 1 Department of Psychiatry, Bellvitge University Hospital-IDIBELL, Barcelona, Spain; 2 Ciber Fisiopatología Obesidad y Nutrición (CIBERObn), Instituto de Salud Carlos III, Madrid, Spain; 3 Departament de Psicobiologia i Metodologia de les Ciències de la Salut, Universitat Autònoma de Barcelona, Barcelona, Spain; 4 Department of Clinical Sciences, School of Medicine, University of Barcelona, Barcelona, Spain; 5 CIBER Salud Mental (CIBERSAM), Instituto de Salud Carlos III, Madrid, Spain; 6 Yale University School of Medicine, Department of Psychiatry, New Haven, CT USA; 7 Yale University School of Medicine, Department of Neuroscience, New Haven, CT USA; 8 Yale University School of Medicine, Yale Child Study Center, New Haven, CT USA; 9 Yale University School of Medicine, The National Center on Addiction and Substance Abuse, New Haven, CT USA; 10 Connecticut Mental Health Center, New Haven, CT USA; 11 Melbourne School of Psychological Sciences, University of Melbourne, Parkville, Victoria Australia; 12 Universidad Internacional de la Rioja, la Rioja, Spain

**Keywords:** Psychology, Human behaviour

## Abstract

Impulsivity is a multidimensional construct. Although gambling disorder (GD) has been associated with high impulsivity, impulsivity across multiple domains has not been thoroughly investigated in this population. We first aimed to examine whether associations between three facets of impulsivity (response impulsivity, choice impulsivity and impulsive tendency) varied between GD patients and healthy controls (HC). We next aimed to evaluate relationships between these three types of impulsivity, as proposed by theoretical models of impulsivity, and their associations with GD severity. The sample included 97 treatment-seeking adult men with GD, diagnosed according to DSM-5 criteria, and 32 male HCs recruited from the general population. Greater impulsivity in all three domains was found in men with GD in comparison to men without GD. Associations between impulsivity facets were found in both groups, with response impulsivity being the only domain associated with GD severity. Our findings confirm that multiple domains of impulsivity are relevant in GD. Future studies should examine the extent to which treatments aimed at targeting specific aspects of impulsivity improve outcomes.

## Introduction

Although impulsivity has been proposed as a multifactorial construct^1^, there is still a lack of consensus regarding its definition and the independence of impulsivity domains^2^. Impulsivity has been defined as a tendency to respond with little forethought, often with disregard to the negative consequences to the impulsive individual or others^3^. Impulsivity has been found to factor into multiple forms, including response and choice forms, that can be measured across species^4–7^. While multiple theoretical models have been proposed different types of impulsivity, the proposal by MacKillop *et al*.^8^ is widely used and validated in different populations. This model posits that impulsivity can be partitioned into three main domains: response impulsivity, choice impulsivity and impulsive tendencies.

Response impulsivity, also termed impulsive action or motor impulsivity, involves impairments in delaying, withholding or interrupting inappropriate responses^4,9^. High levels of this type of impulsivity have been associated with gambling disorder (GD), with GD participants demonstrating differences in response impulsivity in comparison with healthy control (HC) participants^10^, including within treatment-seeking samples^11^. Multiple studies suggest gambling severity is positively correlated with motor impulsivity^9,12,13^ and one recent meta-analysis found GD to be associated with significant impairments in motor and attentional inhibition^14^.

Delay discounting relates to impulsive choice and the extent to which an individual prefers a smaller-sooner over a larger-later reward^5,15^. In the case of GD, cognitive disturbances related to risk-reward decision making have been reported^16,17^, and individuals with GD tend to discount rewards more steeply than controls^18–21^. Increasing evidence supports that people with gambling problems prefer to obtain an economic reward in the most immediate way possible that provides them with the sensation that they are winning and able to continue betting, rather than receiving larger amounts of money in a more distant time^22^. However, some research on GD severity and choice impulsivity has been inconsistent. Some studies suggest that GD severity and age may be the best statistical predictors of individual differences in delay-discounting rates^22–24^ and a recent meta-analysis of impulsivity in GD found evidence of a significant publication bias in delay discounting studies in GD populations^14^. However, some others have found that choice impulsivity cannot discriminate between individuals with problematic gambling and those with GD^13,25^. Further, some theoretically related constructs (e.g., learning to make advantageous choices during a risk/reward decision-making task) have found GD to associate with disadvantageous decision-making whereas others have not^26^.

A third form of impulsivity, henceforth termed “impulsive tendency” (also known as impulsivity trait), has been proposed. For example, the Barratt Impulsiveness Scale (BIS-11) has been found to factor into several domains including those relating to motor, non-planning and inattention^27,28^. While the BIS-11 has been studied across diagnostic groups (including in studies linking the measure to biological measures like brain structure in GD, drug addiction and non-addicted states), concerns have been raised regarding inconsistencies in factor structure across studies^29^, including within GD samples^30^. On the other hand, the UPPS-P model, derived from the extant literature and updated over time, proposes five factors of impulsivity: (lack of) perseverance, (lack of) premeditation, positive and negative urgency, and sensation-seeking^31,32^. Empirical studies have repeatedly reported an association between GD and impulsive tendencies^33–36^. In particular, higher lack of perseverance, and positive and negative urgency levels have been found to be the features that best distinguish between patients with GD and HC^37,38^. Similarly, lack of premeditation is positively associated with poor decision-making and an inability to identify the possible negative financial outcomes that might stem from taking risks, a relevant feature in patients with GD^20,39^. Other studies highlight that urgency levels, characterized by the tendency to act rashly when experiencing extreme moods, are linked with GD severity and other impulsive behaviors^40,41^. It should also be noted that impulsivity stemming from enduring personality characteristics that lead gamblers to focus on short-term gains (i.e. trait impulsivity) have been found to be more strongly linked to problem gambling rather than momentary cognitive or affective disinhibition (i.e. state impulsivity)^42^.

The existing body of research on impulsivity and GD suggests an association between impulsive response, choice and tendency. Some studies uphold that sensation-seeking, lack of premeditation and urgency could be linked with choice impulsivity and response impulsivity^2^. Similarly, a correlation between impulsive tendencies and choice impulsivity has been described in GD, suggesting that individuals with GD who perceive themselves as being more prone to behaving impulsively may also make impulsive choices^23^. This correlation was also found in another study, but only in young patients with GD^22^. Relatedly, Kräplin *et al*.^43^ found that urgency and premeditation were specifically associated with disadvantageous decision-making. It also remains unclear whether impulsivity levels are associated with treatment outcome. Some research has found high levels of impulsive traits to be associated with relapse and dropout from treatment^40^, whereas other studies have found greater awareness of gambling-related problems to be associated with positive outcomes^44^. Finally, another study found GD to be associated with response impulsivity and choice impulsivity, although only the latter was linked with GD severity^13^.

At present, questions remain regarding relationships between response impulsivity, choice impulsivity, impulsive tendency and gambling. Empirical studies are needed to examine the multidimensional nature of these impulsive phenotypes in greater depth^8^, and whether the interrelatedness of these domains differ between those with GD and those without. As such, the aim of this study was two-fold. Our first aim was to examine whether the associations between three facets of impulsivity (response impulsivity, choice impulsivity and impulsive tendency) varied between GD patients and HC. Our second aim was to evaluate the intercorrelatedness of these three types of impulsivity in GD, and their association with GD severity. We hypothesized that GD, as compared to HC participants, would exhibit greater impulsivity in all three domains, and that response impulsivity, choice impulsivity, and impulsive tendency would correlate with one another to varying degrees in the GD group, and GD severity would relate to impulsivity in the GD group.

## Material and Methods

### Participants and procedure

An initial sample of 193 patients diagnosed with GD from the Department of Psychiatry at our University Hospital, consecutively recruited between September 2017 and April 2018, was included in the study. Only patients who sought treatment for GD as a primary mental health concern and who met DSM-5 GD criteria^45^ were included. Patients were voluntarily referred to our GD Unit through general practitioners or via other healthcare professionals.

Regarding sociodemographic features, data suggest a negative correlation between impulsivity and chronological age^46,47^, and higher impulsivity levels in males^48^. For this reason, in the present study, male participants aged between 18 and 50 years were included (which define the range of young- to middle-age adulthood). Into the research area of gambling related problems, the definition of age thresholds for elderly substantively vary, being the lower bound usually between age 50+ to 70+ years across researches^49^. In fact, most studies outline that it is precisely the transition from middle-age adulthood to older age (around 50 years-old) the critical phase with relevant adjustments and changes which can significantly affect the gambling habits, being the most relevant risk factors for GD some socio-demographical variations (e.g. employment retirement, financial disadvantages or social isolation)^50,51^, the age-related neurological vulnerabilities in the mechanisms related with behavioral regulation and diminished executive functioning typical of elderly^52–54^, and the physical and psychological unhealthy typical of the senior age (such as chronic medical conditions, limited mobility, anxiety or depression)^55^.

From this sample, 96 cases were excluded because they did not meet the inclusion criteria for this study: they were over 50 (n = 42), suffered from a comorbid mental disorder (i.e. schizophrenia or other psychotic disorders) (n = 17), did not meet DSM-5 criteria for GD (n = 5), were female (n = 22), or could not participate for practical reasons (n = 10). The final sample was made up of 97 treatment-seeking adult men. No comorbid conditions characterized by high levels of impulsivity were found in the study sample, except for substance use disorder (reported by n = 8 GD patients).

Experienced psychologists and psychiatrists conducted face-to-face clinical interviews to assess clinical and demographic variables, such as education level, origin or civil status. Patients were diagnosed with GD according to DSM-5 criteria^45^.

Participants, before initiating outpatient treatment, individually completed all the questionnaires utilized in this study. Neuropsychological measures were completed under the supervision of a staff psychologist on the same day as the rest of the assessment.

Our study sample also incorporated 32 HC participants recruited using word of mouth. The exclusion criteria for the HC group included a lifetime history of GD, being female (to avoid introducing bias in the study design) or not being within the established age range (between 18 and 50 years, inclusive). The comparison group was recruited from the surrounding community. The evaluation protocol was identical to that of the clinical group in that the participants were all evaluated on the same day.

### Measures

#### GD severity

DSM-5 Criteria^45^. Patients were diagnosed with gambling disorder if they met DSM-5 criteria^45^, which consist of nine different criteria and the presence of the disorder is set at a cut-off point of 4 or more.

South Oaks Gambling Screen (SOGS). This self-report 20-item screening questionnaire discriminates between probable pathological, problem and non-problem gamblers^56^. The Spanish validation used in this work showed excellent internal consistency (α = 0.94) and test–retest reliability (r = 0.98)^57^.

#### Response impulsivity

Conners’ Continuous Performance Test, 2nd edition (CPT-II). The CPT-II is a computer-based task that involves participants pressing the space bar in response to visual stimuli (i.e., letters on a computer screen) that are presented over a span of 14 min^58^. The CPT-II provides information about the participants’ omission and commission error rates, reaction time, and response variability, which represent an assessment of sustained attention and inhibitory control. Higher scores on the CPT-II indicate worse performance.

#### Choice impulsivity

Delay discounting task. This 27-item self-administered tool was used to measure individual inter-temporal discount rates (k), providing a set of alternative choices between a smaller, immediate monetary reward and a larger, delayed monetary reward^59^. Each question was designed to correspond to a different k value, which constitutes the measure of discounting rate and represents the amount of discounting of the later reward that renders it equal to the smaller reward. Respondents’ answers are placed on reference discounting curves, where placement amid steeper curves indicates higher levels of choice impulsivity. Single k parameter-estimates can be obtained not only for an overall rate of discounting, but also for items with small, medium and large monetary rewards^59^. K values can range from 0 (selection of the delayed reward option for all items, or no discounting) to 0.25 (selection of the immediate reward option for all items, or always discounting). According to many studies using the delay discounting task (also termed the Monetary Choice Questionnaire)^22,60^, the distributions of k values were normalized using square root transformation.

#### Impulsive tendencies

Impulsive Behavior Scale (UPPS-P). The UPPS-P measures five facets of impulsive behavior through self-report on 59 items: negative urgency; positive urgency; lack of premeditation; lack of perseverance; and sensation-seeking^32^. The Spanish-language adaptation of the UPPS-P showed good reliability (Cronbach’s α between 0.79 and 0.93) and external validity^61^.

### Ethics

The present study was carried out in accordance with the latest version of the Declaration of Helsinki. The University Hospital Clinical Research Ethics Committee approved the study, and signed informed consent was obtained from all participants.

### Statistical analysis

Statistical analyses were conducted with Stata15 for Windows. The comparison between the impulsivity measures between the groups (HC versus GD) was based on analysis of variance adjusted for the participants’ ages, education levels and presence of substances use (ANCOVA). Associations between variables (impulsivity measures and GD severity measures) were estimated through partial correlation coefficients, also adjusted for age, and education and substance use. In addition to the correlational analysis, multiple regression models were used to obtain a predictive model of the GD severity (defined as the dependent variable) based on impulsivity measures. These models were generated in two step/blocks: (a) first block entered and set the covariates age and education level; (b) the second block automatically selected the significant contributors to GD severity from the impulsivity measures through a stepwise procedure. The incremental predictive capacity of impulsivity on the criteria was valued with change/increase in the R^2^ coefficient.

In this study, effect sizes for mean comparisons were obtained through Cohen’s d coefficient, considering 0.5 > |d| > 0.20 to be a small effect, 0.8 > |d| > 0.5 to be a moderate effect and |d| > 0.8 to be a large effect^62^. In addition, and due to the strong association between the sample size and significance tests for correlation estimates, 0.24 > |r| > 0.10 was considered to be small, 0.37 > |r| > 0.24 to be medium and |r| > 0.37 to be large (these thresholds corresponds to Cohen’s d values of 0.20, 0.50 and 0.80 respectively^63^.

Finally, increases in the type-I error due to multiple comparisons was controlled using the Finner method, a procedure included in family-wise-error-rate stepwise systems, which has been reported to be more appropriate than Bonferroni correction^64^.

## Results

### Sample description

The mean age for the HC group was 31.3 years old (SD = 6.6). Most participants had completed secondary school (53.1%), 37.5% had a university education and 9.4% a primary school level of education. Most were born in Spain (87.5%) and were employed (71.9%).

The mean age for the GD group was 35.0 years (SD = 8.8). Most had a primary school level of education (55.7% versus 40.2% secondary school and 4.1% university). Most were born in Spain (87.6%) and were employed (72.2%).

Significant differences were found between groups in terms of education level (χ^2^ = 34.2, df = 2, p < 0.001) and age (T = 2.2, df = 128, p < 0.030). Thus, we controlled for these factors in subsequent between-group analyses. No differences between the groups were found for marital status (χ^2^ = 4.58, df = 2, p = 0.106), immigration status (χ^2^ = 0.01, df = 1, p = 0.985) and employment status (χ^2^ = 0.02, df = 1, p = 0.975).

### Comparisons between the groups: ANCOVA

ANCOVAs confirmed that the GD group demonstrated greater GD severity than the HC group (Table 1). The GD group also demonstrated more commission errors on the CPT, demonstrated steeper discounting rates, and scored higher on all UPPS-P subscales (Table 1).

**Table 1:** Comparison between the groups: ANCOVA.

	α	Control *n* = 32		Gambling Disorder *n* = 97		*F*	*df*	*p*	*|d|*
		Mean	SD	Mean	SD				
*Gambling severity*									
DSM-5 total criteria	0.934	0.06	0.25	7.24	1.66	591.45	1/127	<0.001*	**6.05**^**†**^
SOGS total score	0.822	0.22	0.42	11.44	2.85	490.21	1/127	<0.001*	**5.51**^**†**^
^1^*Response impulsivity, CPT*									
Omissions		2.09	1.76	2.29	5.73	0.03	1/125	0.868	0.05
Commissions		12.78	7.29	21.78	16.78	6.43	1/125	0.012*	**0.70**^**†**^
Hit Reaction Time		388.72	38.71	392.59	331.46	0.01	1/125	0.955	0.02
Perseveration		−0.03	0.74	1.18	4.07	2.06	1/125	0.154	0.41
^1^*Delay discounting*									
K, overall square root		0.1360	0.0782	0.1997	0.1553	3.91	1/125	0.047*	**0.52**^**†**^
^1^*Impulsivity tendency, UPPS-P*									
Lack of premeditation	0.872	20.33	4.80	24.69	6.59	8.64	1/125	0.004*	**0.76**^**†**^
Lack of perseverance	0.783	17.68	4.37	22.69	4.99	18.55	1/125	<0.001*	**1.07**^**†**^
Sensation-seeking	0.864	25.39	8.20	30.04	8.47	5.56	1/125	0.043*	**0.56**^**†**^
Positive urgency	0.942	20.06	5.92	32.24	10.47	28.10	1/125	<0.001*	**1.43**^**†**^
Negative urgency	0.909	20.77	5.72	32.89	7.76	47.75	1/125	<0.001*	**1.78**^**†**^

*Note*. SQRT: Square root. SD: standard deviation. α: Cronbach’s alpha in the sample.

^1^Results adjusted for age and education levels.

*Bold: significant comparison (0.05 level). ^†^Bold: effect size in the moderate (*|d|* > 0.50) to high range (*|d|* > 0.80).

*p*-values include Finner’s correction for multiple comparisons.

### Associations between impulsivity measures

The upper part of Table 2 contains the correlation matrix (partial correlations adjusted for age, education level and substance use) measuring associations between the impulsivity measures in the GD group. Positive coefficients in the moderate to high range were obtained between choice impulsivity and CPT-related omissions and perseveration and between the UPPS-P positive urgency and negative urgency measures. Positive associations were also obtained between most UPPS-P subscales.

**Table 2:** Associations between impulsivity measures: partial correlations adjusted for age and education level.

		1	2	3	4	5	6	7	8	9	10
1	CPT omissions	—	0.03	−0.04	**0.47**^**†**^	**0.28**^**†**^	0.07	0.06	0.09	0.22	0.12
2	CPT commissions	***0.37***^***†***^	—	−0.07	0.03	0.01	0.04	0.04	0.01	0.09	0.02
3	CPT hit reaction time	−*0.07*	−***0.61***^***†***^	—	−0.02	0.14	0.07	0.06	−0.09	−0.03	−0.03
4	CPT perseveration	***0.36***^***†***^	***0.45***^***†***^	−*0.11*	—	**0.24**^**†**^	−0.05	0.01	0.02	0.11	0.06
5	k-Overall square root	*0.10*	−*0.15*	***0.27***^***†***^	−*0.07*	—	0.20	0.13	0.08	**0.37**^**†**^	**0.27**^**†**^
6	UPPS-P Premeditation	*0.14*	*0.09*	−*0.06*	*0.17*	***0.33***^***†***^	—	**0.47**^**†**^	−0.08	0.20	**0.24**^**†**^
7	UPPS-P Perseverance	*0.13*	***0.30***^***†***^	−***0.40***^***†***^	*0.18*	*0.04*	***0.45***^***†***^	—	−0.19	0.15	**0.25**^**†**^
8	UPPS-P Sensation	−*0.02*	−*0.03*	*0.15*	*0.08*	*0.09*	*0.20*	−*0.11*	—	**0.36**^**†**^	**0.32**^**†**^
9	UPPS-P Positive Urge	*0.16*	*0.23*	−*0.13*	***0.40***	***0.43***^***†***^	***0.35***^***†***^	*0.17*	*0.06*	—	**0.76**^**†**^
10	UPPS-P Negative Urge	−*0.05*	*0.09*	−***0.34***^***†***^	*0.03*	***0.26***^***†***^	***0.37***^***†***^	***0.24***^***†***^	−*0.08*	***0.64***^***†***^	—

Note.^†^Bold: effect size in the moderate (|r| > 0.24) to high range (|r| > 0.37).

Upper part of the table: correlations estimated in the GD group (*n* = 97).

Lower part of the table, italic font: correlations estimated in the HC group (*n* = 32).

The lower part of Table 2 contains the correlation matrix for the HC group. In this subsample, choice impulsivity positively correlated with the CPT hit-reaction-time, and with the UPPS-P premeditation, positive urgency and negative urgency measures. Other correlations emerged: (a) CPT commissions positively correlated with UPPS-P perseverance; (b) CPT hit-reaction time negatively correlated with UPPS-P perseverance and negative urgency measures; (c) CPT perseverance positively correlated with UPPS-P positive urgency; and, (d) CPT measures correlated with one another (except for perseveration and hit-reaction-time), as well as several UPPS-P measures (Table 3). Choice impulsivity measures correlated with UPPS-P measures of premeditation and positive and negative urgency. Positive associations were also obtained between many UPPS-P subscales, and between CPT omissions and commissions measures.

**Table 3:** Associations between impulsivity and GD: partial correlations adjusted for age and education level.

	GD group (*n* = 97)										HC group (*n* = 32)	
	Total score		^1^DSM-5 criteria								Total score	
	DSM-5	SOGS	C1	C2	C3	C4	C5	C6	C7	C8	DSM-5	SOGS
*Response impulsivity, CPT*												
Omissions	0.11	0.16	0.18	0.07	−0.06	0.10	0.03	0.02	0.10	−0.10	−0.05	0.13
Commissions	0.11	0.09	0.10	0.08	0.00	0.03	**0.24**^**†**^	0.02	−0.07	0.04	**0.40**^**†**^	0.18
Hit Reaction Time	0.10	−0.07	0.07	0.05	0.03	0.06	0.05	0.04	0.04	0.04	−**0.35**^**†**^	−0.09
Perseveration	0.01	0.08	0.15	0.08	0.02	−0.06	−0.16	0.07	0.05	−0.25	−0.12	−0.21
*Choice impulsivity, DELAY*												
SQRT_K-Overall	0.19	**0.35**^**†**^	**0.24**^**†**^	0.12	−0.04	**0.30**^**†**^	0.03	−0.05	0.12	−0.06	−0.05	**0.44**^**†**^
*Impulsivity tendency, UPPS-P*												
Lack of premeditation	**0.27**^**†**^	0.21	0.10	0.10	0.14	0.22	0.19	−0.08	0.12	0.18	−0.06	−0.02
Lack of perseverance	**0.36**^**†**^	**0.34**^**†**^	**0.30**^**†**^	**0.40**^**†**^	**0.24**^**†**^	0.12	0.18	−0.08	0.14	0.07	**0.34**^**†**^	0.04
Sensation seeking	0.21	0.16	**0.25**^**†**^	0.14	0.08	0.04	0.20	0.10	−0.13	0.05	−0.20	0.01
Positive urgency	0.17	0.22	**0.30**^**†**^	**0.32**^**†**^	0.19	**0.28**^**†**^	0.23	−0.04	0.12	−0.02	0.05	**0.24**^**†**^
Negative urgency	0.16	0.05	0.15	**0.26**^**†**^	**0.25**^**†**^	**0.24**^**†**^	**0.26**^**†**^	−0.08	0.01	−0.06	0.15	0.16

*Note*. SQRT: Square root. ^†^Bold: effect size in the moderate (*|r|* > 0.24) to high range (*|r|* > 0.37).

^1^DSM-5 criteria for GD: C1 = gamble with increasing amounts of money, C2 = irritability, C3 = unsuccessful efforts to control, C4 = preoccupations, C5 = gamble as a way of escaping, C6 = after losing returns other days, C7 = lies related to gambling activity, C8 = social impairment. ^1^Point-biserial correlation estimates.

### Associations between impulsivity and GD severity

**Figure 1:**
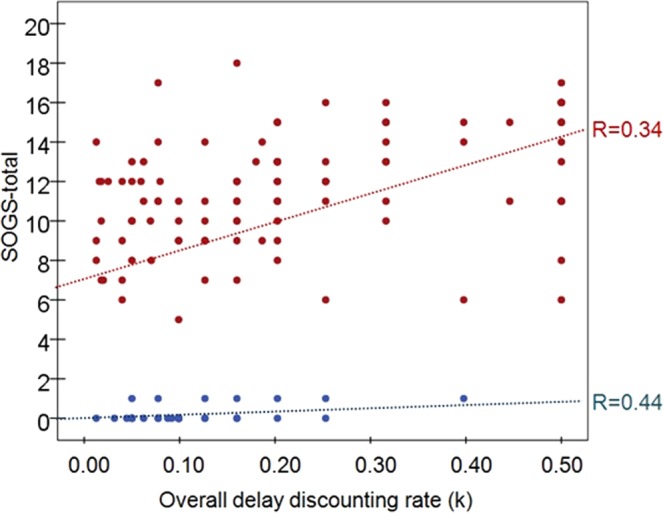
Scatter-plot showing relationship between delay discounting and GD severity. Note. R: Pearson correlation coefficient. Red color: GD group (n = 97). Blue color: HC group (n = 32).

The first block of Table 3 contains the partial correlations (adjusted for age, education levels and substance use) between impulsivity measures with GD severity (total DSM-5 criteria and SOGS total) and each DSM-5 criteria in the GD group (point-biserial correlations were obtained for examining the relationships between impulsivity measure with each DSM-5 criterion). Results show that the DSM-5 total criteria for GD positively correlated with UPPS-P lack of premeditation and lack of perseverance, while the SOGS total score was positively associated with delay discounting and UPSS-P lack of perseverance. Figure 1 contains the scatterplot between overall delay discounting scores (k) and SOGS scores. Regarding the association between the impulsivity measures with each DSM-5 criteria for GD, choice impulsivity positively correlated with criterion 1 “gamble with increasing amounts of money” and criterion 4 “preoccupied for gambling activity”, CPT commissions score was related to criterion 5 “gambling as a way of escaping” and impulsivity tendency measured with the UPPS-P scales tended to correlate with criterion 1 to 5.

In the HC group (second block of Table 3), DSM-5 total GD criteria positively correlated with CPT commissions and with the UPPS-P lack of perseverance; in this group, the SOGS total score positively correlated with measures of delay discounting and UPSS-P positive urgency. The matrix correlation was not obtained for impulsivity with each DSM-5 criteria in this group due the extremely low prevalence for the gambling symptoms in this subsample.

Table 4 contains the predictive regression models for GD severity based on the impulsivity measures and adjusted for age and education levels in the GD group. The first model was obtained for the number of DSM-5 criteria for GD, and adjusted for age and education level. The significant predictors of higher severity were lack of perseverance and positive urgency. The second model, obtained for the SOGS total score, retained as significant predictors the delay impulsivity, lack of perseverance, sensation seeking and positive urgency.

**Table 4:** Predictive model for the GD severity: multiple regressions adjusted for age and educational level in the GD group.

Criterion: DSM-5 total criteria	B	SE	Beta	T	p	95%CI B		ΔR^2^
*First step/block (covariates)*								0.009
Age (years-old)	0.012	0.020	0.063	0.605	0.547	−0.027	0.051	
Education level	−0.088	0.140	−0.066	−0.630	0.530	−0.367	0.190	
*Second step/block*								0.222
Age (years-old)	0.019	0.017	0.103	1.101	0.274	−0.015	0.054	
Education level	−0.158	0.126	−0.118	−1.255	0.213	−0.409	0.092	
UPPS-P Lack of perseverance	0.100	0.031	0.301	3.182	0.002	0.038	0.162	
UPPS-P Positive urgency	0.052	0.015	0.325	3.492	0.001	0.022	0.081	
Criterion: SOGS total	B	SE	Beta	T	p	95%CI B		ΔR^2^
*First step/block (covariates)*								0.025
Age (years-old)	0.042	0.033	0.130	1.261	0.210	−0.024	0.108	
Education level	−0.175	0.240	−0.075	−0.728	0.468	−0.651	0.301	
*Second step/block*								0.270
Age (years-old)	0.063	0.031	0.196	2.070	0.041	0.003	0.124	
Education level	−0.298	0.215	−0.129	−1.386	0.169	−0.726	0.130	
Delay: SQRT_K-Overall	5.987	1.716	0.326	3.488	0.001	2.577	9.397	
UPPS-P Lack of perseverance	0.229	0.056	0.399	4.105	<0.001	0.118	0.340	
UPPS-P Sensation seeking	0.095	0.035	0.279	2.728	0.008	0.026	0.163	
UPPS-P Positive urgency	−0.082	0.037	−0.222	−2.183	0.032	−0.156	−0.007	

*Note*. SQRT: Square root. GD group (*n* = 97).

## Discussion

The present study analyzed whether associations between response impulsivity, choice impulsivity and impulsive tendency varied between GD patients and HC. Moreover, the interrelationship among these three types of impulsivity, and their associations with GD severity in GD, were examined. Our hypotheses were largely supported, and the implications of the findings are discussed below. In the present study, 8.24% of the sample included also presented substance abuse. This comorbidity has been recorded as the most frequent in gamblers^65^. In contrast, other disorders related to gambling behavior and impulsivity, such as eating disorders^66^, or impulse control disorders^67^, were not found in the current sample.

Regarding response impulsivity, patients with GD reported greater commission errors, defined as incorrect responses towards non-target stimulus, in comparison with HC participants. This result dovetails with previous studies finding that patients with GD are more prone to commit execution errors when facing no-go stimuli^9^ and a recent meta-analysis identifying motor impairments in individuals with GD^14^. This leads us to postulate that response impulsivity is linked to a deficit in inhibitory control, which could partially explain difficulties in reducing or eliminating gambling behavior.

The findings of this study also showed that, in terms of choice impulsivity, patients with GD presented greater delay discounting than did HC participants. This finding is consistent with other studies highlighting that patients with GD differ from HC when making monetary decisions, showing a biased tendency to discount rewards more rapidly and to select smaller, sooner amounts of money^18^. These results may partly relate to why patients with GD may choose bets for more immediate gains, despite the negative consequences that such gambling may entail^19^.

Furthermore, higher levels of all assessed dimensions of impulsive tendencies were observed in the GD relative to the HC group. This result is partially in line with previous studies that found higher impulsive tendencies, although results have varied between groups in sensation-seeking tendencies^37^ and lack of perseverance^38^. These differences between groups could be explained due, to some extent, to the strong associations between these impulsive dimensions and essential GD clinical features, such as cognitive distortions^37,68,69^, gambling choices^70,71^, emotion-regulation impairment^72^, or GD-related illegal acts^34^. Sensation seeking as well as urgency, which appear to be related with GD severity in some studies, including ours^36,38,73^, are strongly associated with emotional factors^74^. It is well known that difficulties in recognizing and dealing with different emotions are risk factors for the onset and maintenance of GD^75,76^. With respect to the predictors of greater severity, positive urgency and lack of perseverance were found to be the most related when assessed with the DSM-5 criteria^45^. Nevertheless, regarding the SOGS^56^, the most notable dimensions were positive urgency, sensation seeking and lack of perseverance. Similarly, Savvidou *et al*. (2017)^77^ found that in a large sample of men and women significant severity predictors were negative urgency and sensation seeking. Furthermore, a recent study^69^ found that lack of perseverance predicted treatment dropout, and negative urgency was linked to relapses. Regarding the lack of a predictive relationship of the negative urgency and severity of the disorder, it could be hypothesized that negative urgency, that is, gambling when feeling negative emotions, is more associated with female gamblers^78,79^, and that, in general, it may be a factor more linked to the maintenance of the gambling behavior rather than to the severity of the disorder.

Another finding to emerge from the present study is the association between the three impulsivity domains. Considering the clinical group, our results identified an association between choice impulsivity and impulsivity tendencies, with urgency being the dimension which had the greatest association with delay discounting. This finding is consistent with earlier studies highlighting a significant correlation between these two impulsivity facets^22,37^. This is in contrast to the HC group, which presented a significant association between delay discounting and lack of premeditation. Our results also indicate a positive correlation between response impulsivity, as assessed on the CPT, and choice impulsivity. This finding seems to be partially consistent with other research which found weak or no relationships between most facets of response and choice impulsivity^8,80^. The finding that there are several domains of response and choice impulsivity is consistent with the multifactorial frameworks of impulsivity.

Finally, a significant association was found between GD severity and two of the impulsivity facets, impulsive tendencies and choice impulsivity, which is consistent with other findings^24,40^. Even though a recent meta-analysis identified associations between motor impulsivity and GD^9^, our study failed to identify a significant association between response inhibition and GD severity among GD patients. This finding suggests that choice impulsivity, impulsive tendencies and response impulsivity could be considered as three separable entities, although the former two in particular seem to be partly inter-related. However, an impaired ability to inhibit motor responses does seem to be associated with greater disorder symptomatology in GD.

From a clinical perspective, it could be postulated that the results of the present study may inform potential treatment targets in the future. Specific adjuvant interventions to address the facets of impulsivity associated with GD severity could potentially improve treatment outcomes^4,5^. In this sense, technologically based interventions represent a new frontier for treatment, from the computerized adaptation of neurocognitive tasks to evaluate these processes, such as cognitive and attentional bias^81^, to the use of mobile applications to condition the selection of healthy foods obesity^82^, or serious games for the treatment of impulsivity in gambling disorder^83^, in eating disorders^84,85^ and in other mental disorders^86^. It has been observed that the use of therapeutic video games, as an additional therapeutic tool can treat difficulties in emotional regulation and impulsivity^84^. Moreover, this modality of treatment increases the motivation of the patient and decreases dropout rates^83^.

### Limitations and future research

The results of this study should be considered in light of its limitations. First, the sample was entirely male. Some studies carried out in healthy participants have found gender-related differences in impulsive tendencies^87^, choice impulsivity^88^ and response impulsivity^89^. Future studies would benefit from including women and comparing both groups from a three-factor impulsivity perspective^8^. Second, the number of patients with GD in the present study was higher than the number of HC participants and there was a lack of group matching on the demographic measures. Future studies should include larger and more balanced HC samples. Third, both delay discounting and impulsive tendencies were evaluated through self-report assessments, and self-report and behavioral measures of impulsivity (even within the same domain) may weakly correlate or be uncorrelated^90^. Further, the extent to which these self-report measures may relate to decision-making processes that may be sensitive to contextual factors and may involve irrational and spontaneous aspects requires additional investigation. Relatedly, although the differences found in the present study would be also related with differences in baseline general IQ^91^, the present study did not measure, report or control for IQ scores. Fourth, the results should be interpreted cautiously given that separate instruments (some self-report and some behavioral) were used to evaluate each type of impulsivity, and poor concordance between self-report and behavioral measures of impulsivity has been reported^92^. The same could be argued for the measures used to assess gambling severity. The DSM only provides a measure for the absence or presence of gambling symptoms and does not take factors such as frequency or breadth of gambling activities into account. Differing findings between the SOGS and the DSM with regards to impulsivity measures could be attributed to this fact. Fifth, previous research has suggested that, in the case of choice impulsivity, delay discounting levels in people with GD vary according to whether they are in a gambling context or not^93^. Relatedly, some individuals with GD may have a contextual control over discounting, choosing delayed rewards in order to avoid spending money immediately through gambling behavior^94^.

Future research should examine facets of impulsivity in different contexts and in relation to individual differences in gambling-related cognitions^68^, in order to obtain a more precise evaluation of how different aspects of impulsivity relate to gambling behaviors. Moreover, validated instruments were not used to screen psychiatric morbidities in the HC group. Longitudinal research is needed to understand changes in impulsivity over the course of addiction, particularly as changes in impulsivity may relate importantly to treatment outcomes^11,95^. Finally, this study was carried out with the aim to assess the specific contribution of impulsivity domains on GD, and therefore other comorbid conditions were excluded from the clinical and control subsamples. Other studies should address what is the impulsivity pattern in heterogeneous samples of patients with GD but also other comorbidities, particularly those clinical conditions that scientific literature has strongly related to impulsivity. Future research also should include comprehensive methods for accurately testing, screening and diagnosing the presence of these other comorbid mental problems, with the aim to know the specific contribution of impulsivity in each condition as well as the interactions between them.

## Conclusions

Taken together, one of the more significant findings to emerge from this study is the confirmation that impulsivity is not a singular construct in the case of GD and these domains of impulsivity are intercorrelated. In the study at hand, we studied three different impulsivity domains: choice impulsivity, trait impulsivity and motor impulsivity and the first two appear to be interrelated. The current data also highlight the interrelationship between these impulsivity facets and GD severity, suggesting that motor response impulsivity is not directly associated with GD severity. Assessing multiple facets of impulsivity within an individual subject complements recent research highlighting the pooled effects of multiple studies identifying robust deficits in multiple cognitive domains in individuals with GD^14^ and offers a path forward for research examining candidate cognitive vulnerability features in gambling populations.
